# Cryopreservation of bioflavonoid-rich plant sources and bioflavonoid-microcapsules: emerging technologies for preserving bioactivity and enhancing nutraceutical applications

**DOI:** 10.3389/fnut.2023.1232129

**Published:** 2023-09-14

**Authors:** Jia Xiang, Ronald Mlambo, Ibrahim Shaw, Yimer Seid, Hamid Shah, Yongju He, Julius K. S. K. Kpegah, Songwen Tan, Wenhu Zhou, Binsheng He

**Affiliations:** ^1^Academician Workstation, Changsha Medical University, Changsha, China; ^2^Xiangya School of Pharmaceutical Sciences, Central South University, Changsha, Hunan, China; ^3^School of Materials Science and Engineering, Central South University, Changsha, Hunan, China; ^4^Department of Plastic Surgery, Third Xiangya Hospital, Central South University, Changsha, Hunan, China

**Keywords:** bioflavonoids, cryopreservation, microencapsulation, antioxidant, anticancer

## Abstract

Bioflavonoids are natural polyphenolic secondary metabolites that are medicinal. These compounds possess antitumor, cardioprotective, anti-inflammatory, antimicrobial, antiviral, and anti-psoriasis properties to mention a few. Plant species that contain bioflavonoids should be preserved as such. Also, the bioactivity of the bioflavonoids as neutraceutical compounds is compromised following extraction due to their sensitivity to environmental factors like light, pH, and temperature. In other words, the bioflavonoids’ shelf-life is affected. Scientists noticed that bioflavonoids have low solubility properties, poor absorption, and low bioavailability following consumption. Researchers came up with methods to encapsulate bioflavonoids in order to circumvent the challenges above and also to mask the unpleasant order these chemicals may have. Besides, scientists cryopreserve plant species that contain bioflavonoids. In this review, we discuss cryopreservation and bioflavonoid microencapsulation focusing mainly on vitrification, slow freezing, and freeze-drying microencapsulation techniques. In addition, we highlight bioflavonoid extraction techniques, medicinal properties, challenges, and future perspectives of cryopreservation and microencapsulation of bioflavonoids. Regardless of the uniqueness of cryopreservation and microencapsulation as methods to preserve bioflavonoid sources and bioflavonoids’ bioactivity, there are challenges reported. Freeze-drying technology is costly. Cryoprotectants damage the integrity of plant cells, to say the least. Researchers are working very hard to overcome these challenges. Encapsulating bioflavonoids via coaxial electrospray and then cryopreserving the micro/nanocapsules produced can be very interesting.

## 1. Introduction

Bioflavonoids are polyphenolic-structured secondary metabolites that are synthesized by plants. It has been scientifically proven that bioflavonoids are found in different parts of plants like fruits, leaves, stems, flowers, roots, bark, and so on (1–3). In plants, bioflavonoids serve as protectants. These chemicals protect plants from both biotic and abiotic stress. In addition, bioflavonoids are responsible for the color and aroma plants have. Furthermore, it is reported that these polyphenolic compounds act as UV filters, signaling molecules, phytoalexins, detoxifying agents, and allopathic chemicals among others. As if that is not enough, bioflavonoids help plants acclimatize to heat at the same time while aiding plants to be inured to very low temperatures (freezing tolerance) (2). Scientists manipulated these protective properties of the bioflavonoids and started examining if these chemicals have medicinal properties in animals and humans (Table 1). Evidence is piling up that bioflavonoids have cardioprotective, neuroprotective, nephroprotective, antiviral, antibacterial, antioxidant, hepatoprotective, anti-inflammatory, and anticancer properties among others (3–8).

**Table 1:** Common dietary bioflavonoid food sources and their chemical compositions.

Bioflavonoids class	Food source(s)	Common dietary flavonoids	Health benefits	References
Flavones	Hot peppers, parsley, celery, thyme	Apigenin, Baicalein, Chrysin, Luteolin	Stimulation of antioxidant biomolecules like superoxide dismutase, glutathione reductase, glutathione peroxidase, and catalase. Wound healing, neuroprotective, antidiabetic, anticancer, anti-psoriasis, antioxidant, anti-inflammatory.	(21, 46–48, 78–80)
Isoflavones	Legumes, soybeans, soy foods	Glycitein, Formononetin, Daidzein, Biochanin A, Genistein	Antioxidant, anticancer, antibacterial, antiviral, anti-inflammatory, antidiabetic, cardioprotective, and anti-osteoporosis properties. Treat hormonal disorders.	(63, 64, 81, 82)
Flavanones	Oranges, grapefruits, lemons (citrus fruits)	Hesperetin, Naringenin, Eriodictyol	Antimicrobial, antioxidant, anti-inflammatory, antiviral, neuroprotective, anti-psoriasis	(52–54, 56, 83, 84)
Flavonols	Onions, teas, scallions, berries, kale, apples, broccoli	Quercetin, Isorhamnetin, Myricetin, Kaempferol	Anti-diabetic, anticancer, antioxidant, antimicrobial, antiviral, anti-inflammation, and cardioprotective properties	(59, 78, 85–89)
Flavanols	Green tea, white tea, oolong tea, apples, berries, cocoa-products	Catechin, epicatechin, gallocatechin, epigallocatechin	Antioxidant, reproductive system therapeutic effects, anti-inflammatory, antitumor, cardioprotective effects, anti-obesity properties, neuroprotective properties	(37, 39, 41, 42)
Anthocyanidins	Red wine, berries (blue, red, and purple), grapes (blue and purple)	Delphinidin, Pelargonidin, Cyanidin, Malvidin, Petunidin, Peonidin	Anticancer, antidiabetic, neuroprotective, anti-inflammatory, and antipsychotic properties	(71)
Chalcones	Apples, vegetables, tomatoes, shallots, potatoes, bean sprouts	Chalconaringenin, phloridzin, phloretin, arbutin	Antibacterial, antifungal, antioxidant, antimalarial, antitumor, and antispasmodic properties	(29, 30, 32–34, 90)

Preservation of plant species that are rich in bioflavonoids is of paramount importance. Bioflavonoids have a variety of medicinal properties. One of the biggest advantages of plant-derived medicine is its cost-effectiveness. Medicine should be readily available and cheap. The only way of achieving the former, which automatically connects with the latter, is by preserving the plants rich in these medicinal chemicals. Different preservation methods and techniques have been employed to preserve plant species and their vital chemical components. Two common preservative methods used are cryopreservation and microencapsulation (9–14).

Recently scientists are considering bioflavonoids as potential therapies for different types of cancers like hepatocellular carcinoma. MicroRNAs (miRs) are thought to play key roles in the development of hepatocellular carcinoma (HCC) by controlling the expression of particular target genes (15). A group of scientists then discovered that by influencing the expression of the microRNA transcriptome, apigenin prevents the growth of hepatocellular carcinoma cells. In another study, it was observed that non-small cell lung cancer cells respond differently to Op18/stathmin signaling when autocrine IL-10 is neutralized *in vitro.* Bioflavonoids are therapeutic potential candidates for lung cancer treatment. Hesperetin was found to treat ovarian cancer by interacting with growth factor receptor 2 (HER2). Rutin has the potential to treat osteosarcoma and scientists reported that *in vitro*, restoring microRNA-130b expression reduces the malignant behavior of osteosarcoma cells. Also, scientists report RNA adenosine modifications related to prognosis and immune infiltration in osteosarcoma (16–24).

## 2. Bioflavonoids and their sources

**Figure 1:**
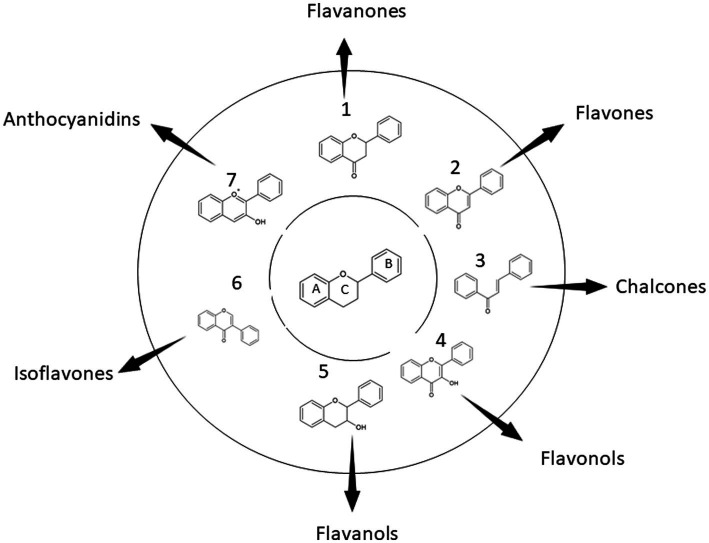
Classification of the main bioflavonoids based on their general structure. Bioflavonoids share a common structure made up of an oxygenated heterocycle (ring C) connected to two aromatic rings (rings A and B) by three carbon atoms. A typical C6-C3-C6 flavan backbone, which is the building block for all flavonoids and comprises two phenyl rings, is produced as a result.

Generally, flavonoids share a common chemical structure skeleton which has three rings. These rings are normally described as C_6_-C_3_-C_6_ and are designated letters A, C, and B, respectively. Modification of the ring brings about variation among the bioflavonoids and hence different subtypes of bioflavonoids. There are seven major subtypes of bioflavonoids (Figure 1): chalcones, flavanols, flavanones, flavones, flavonols, isoflavones, and anthocyanidins. The nomenclature of bioflavonoids is based on the level of oxidation of the middle heterocycle skeleton ring (25–28).

### 2.1. Chalcones

Chalcones are also known as benzyl acetophenones. Scientists describe chalcones as α, β-unsaturated carbonyl compounds, which are ketones. These ketones hold two rings namely A and B. These two rings are interconnected by a 3-carbon atom ketone. In Balkan states, plant species loaded with chalcones had been used as therapeutic agents. These plants include *Ruscus*, *Angelica*, *Piper*, and *Glycyrrhiza* genus. In one research, scientists found out that chalcone induces apoptosis by triggering endoplasmic reticulum (ER) stress through the sulfonation of IRE1α by an enzyme called NADPH oxidase 4 (NOX4). This sulfonation leads to the degradation of mRNA, known as “regulated IRE1α-dependent decay” (RIDD), instead of the typical splicing of XBP1. One consequence of IRE1α sulfonation-induced RIDD is the degradation of miR-23b, which in turn increases the expression of NOX4. The expression of NOX4 is also elevated in breast and prostate cancer tissue. In mouse models treated with chalcone, tumor growth was reduced through the consistent mechanisms involving NOX4-IRE1α sulfonation-RIDD. Additionally, NOX4 activation and IRE1α sulfonation were significantly increased under severe ER stress conditions (29–34).

### 2.2. Flavanols

Flavanols fall under the main subtypes of bioflavonoids. Scientists report that flavanols are found in a variety of food such as cereals, legumes, tea, cocoa, red wine, grapes, fruits, beer, and grapes among others. It is reported that 121 flavanols have been found in plants distributed in 59 species and 29 families. Examples of the species are *Pronephrium penangianum, Camellia sinensis, Theobroma cacao,* and *Astragalus membranaceus*. Green tea(*Camellia sinesis*) catechins are reported to have antioxidant, reproductive system therapeutic effects, anti-inflammatory, antitumor, cardioprotective effects, anti-obesity properties, neuroprotective properties, and so on. In a certain study (35), investigated the effects of catechin on heart function and the expression of a long non-coding RNA called MIAT in two different experimental models: MI/R rats and H/R-induced H9C2 cells. They found that catechin improved heart function in MI/R rats and decreased the expression of MIAT in myocardial tissue. In H/R-induced H9C2 cells, catechin protected against cell apoptosis (programmed cell death), while overexpression of MIAT attenuated this protective effect of catechin. The researchers also discovered that the transcription factor CREB could bind to the promoter region of MIAT, and catechin increased the expression of CREB, resulting in the suppression of MIAT expression. Catechin further enhanced mitochondrial function and reduced apoptosis by activating the Akt/Gsk-3β signaling pathway. Conversely, MIAT inhibited Akt/Gsk-3β activation and promoted cell apoptosis in H/R-induced H9C2 cells. Ultimately, the study concluded that catechin promoted Akt/Gsk-3β activation by inhibiting MIAT expression in H/R-induced H9C2 cells. These findings suggest that catechin has beneficial effects on heart function and mitochondrial function, and its mechanism of action involves the regulation of MIAT and the Akt/Gsk-3β pathway (36–44).

### 2.3. Flavones

Flavones are found in many plant species. The popular genus from which flavones and their derivatives are found in *Scutellaria*. The genus belongs to the mint or *Lamiaceae* family. Plants like mints, lavender, basil, and rosemary belong to this family. It is reported that the *Scutellaria* genus has 360 species, and these species are distributed in East Asia, Europe, and the United States of America (United States). Among the Traditional Chinese Medicine herbs, *Scutellaria baicalensis* (Baikal skullcap) has been used to treat insomnia, diarrhea, dysentery, hypertension, inflammation, respiratory infections, and hemorrhage. Different *in vitro* studies showed that *S. baicalensis* root extracts have anticancer properties in various types of cancer such as myeloma, lymphoma, colon cancer, brain tumor, and lymphocytic leukemia. Recently, scientists (45) discovered that Apigenin exerts its effects by influencing the immune responses mediated by dendritic cells (DCs) and T-cells. The findings indicate that Apigenin shifts the balance of T-cell responses from pro-inflammatory Th1 and Th17 types toward regulatory T-cell responses. This shift is characterized by decreased expression of T-bet and IFN-γ, and IL-17, as well as increased expression of IL-10, TGF-β, and FoxP3. The protein RelB, a key component of the NF-κB pathway, plays a central role in DC maturation, antigen presentation, and DC-mediated T-cell activation. Apigenin was found to reduce the levels of RelB mRNA and protein, as well as inhibit its nuclear translocation. Furthermore, silencing RelB using siRNA enhanced the effects of Apigenin, confirming the involvement of RelB in Apigenin-mediated regulation of DC biology (21, 46–48).

### 2.4. Flavanones

Scientists say that flavanones’ antioxidant properties are attributed to phenolic moieties in their chemical structure (49, 50). The medicinal properties of flavanones are not limited to antioxidant and antimicrobial activities but also include neuroprotective, antidiabetic, anticancer, anti-psoriasis, and so on. The *Erythrina* genus is loaded with flavanones, like the *Erythrina crista-galli*. In 2014, scientists investigated the combined effects of a racemic flavanone called Naringenin (NG) and the protein sericin as blockers of TNF-α. Microparticles of sericin (SMPs) and (R/S) NG-loaded sericin (NGSMPs) were created through spray-drying, and their morphology, particle size distribution, and encapsulation efficiency were examined. The presence of NG did not affect the morphology or particle size distribution of the microparticles. Biological testing was conducted using lipopolysaccharide (LPS)-stimulated human peripheral blood mononuclear cells (hPBMC), comparing the effects of (R/S) NG, SMPs, and NGSMPs. At the highest dose (200 μg/mL), NGSMPs exhibited cytotoxicity, surpassing the effects of (R/S) NG alone. Furthermore, while sericin alone did not effectively reduce LPS-induced serum TNF-α levels, NGSMPs loaded with (R/S) NG (93% encapsulation efficiency) significantly demonstrated greater potency than (R/S) NG alone. In conclusion, this study demonstrated the potential of sericin-based microspheres loaded with TNF-α blockers to regulate this cytokine and presents a foundation for developing new topical formulations for treating mid-stage psoriasis (51–57).

### 2.5. Flavonols

Several *in vivo* and *in vitro* studies showed cardioprotective properties of flavonols, especially quercetin. In addition, medicinal properties such as anti-diabetic, anticancer, antioxidant, antimicrobial, antiviral, and anti-inflammation were also reported. There are many sources of flavonols. Examples of plant species rich in flavonols are *Allium tricoccum* and *Arabidopsis thaliana*. Recently, scientists reported the anti-inflammatory property of quercetin. They investigated the impact of quercetin on macrophages. The results demonstrated that quercetin effectively suppressed the production of various inflammatory molecules, including nitric oxide (NO), interleukin-6 (IL-6), monocyte chemotactic protein-1 (MCP-1), interferon-inducible protein 10 (IP-10), Regulated upon Activation, Normal T Cell Expressed and Presumably Secreted (RANTES), granulocyte-macrophage colony-stimulating factor (GM-CSF), granulocyte colony-stimulating factor (G-CSF), tumor necrosis factor-alpha (TNF-α), leukemia inhibitory factor (LIF), lipopolysaccharide-induced CXC chemokine (LIX), and vascular endothelial growth factor (VEGF). Moreover, quercetin also hindered the release of calcium ions in dsRNA-induced RAW 264.7 mouse macrophages. Additionally, quercetin showed significant inhibition of the mRNA expression of signal transducer and activator of transcription 1 (STAT1) and STAT3 in dsRNA-stimulated RAW 264.7 cells. Collectively, these findings suggest that quercetin possesses beneficial properties in combating viral inflammation by impeding the calcium-STAT pathway, leading to the suppression of NO, cytokines, chemokines, and growth factors in dsRNA-induced macrophages (58–61).

### 2.6. Isoflavones

Isoflavones are commonly found in leguminous plant species like *Medicago truncatula*, *Pisum sativum*, *Phaseolus vulgaris*, *Glycine max*, *Trifolium pratense*, *Trifolium repens*, *Medicago sativa*, and so on. Isoflavone content varies in different plant species. In soybean, for example, isoflavones amount to 1.2 -4.2 mg/dry weight, mainly daidzein, genistein, and their derivatives. Isoflavones are chemoprotective. In addition, isoflavones are used to treat many hormonal disorders, cardiovascular diseases, osteoporosis, and/or menopausal symptoms. Genistein as a member of isoflavones has antioxidant, anticancer, antibacterial, antiviral, anti-inflammatory, and antidiabetic properties. Scientists investigated the impact of genistein, a compound with chemopreventive properties, on the progression of existing prostate cancer. The researchers used transgenic mice with prostatic intraepithelial neoplasia lesions and found that feeding them genistein resulted in a more aggressive advancement of prostate cancer, including increased numbers of poorly differentiated prostates and a higher incidence of pelvic lymph node metastases. The same effects were observed in human prostate cancer cells treated with genistein, which led to enhanced cell proliferation, invasion, and activity of matrix metalloproteinase-9. The researchers also identified osteopontin as a key mediator of genistein’s effects. However, a high pharmacologic dose of genistein counteracted these effects. The study suggests a biphasic regulation of prostate cancer growth and metastasis by genistein, highlighting the need for cautious examination of its effects on hormone-dependent cancers in a chemotherapy context (62–65).

### 2.7. Anthocyanidins

Anthocyanidins are found in many fruits, especially dark-colored fruits. The list of the plant species rich in anthocyanidins is long: *Brassica campestris* L, *Ipomoea batatas* L, *Rheum* spp., *Raphanus sativus* L, *Allium cepa* L, *Solanum tuberosum* L, *Capsicum annuum* L, *Arachis hypogaea* L, *Pisum sativum* L, *Lens culinaris* Medic, *Zingiber officinale*, *Solanum melongena* L, *Daucus carota* L, *Brassica* spp., *Phaseolus* spp., *Asparagus officinalis* L, *Vaccinium myrtillus* L, *Rubus* spp., *Fragaria x ananassa* Duch, and so on. Anthocyanidins have medicinal properties like anticancer, antidiabetic, neuroprotective, anti-inflammatory, antipsychotic, and so on. In one study (66), investigated the cardiovascular effects of malvidin, a polyphenol found in red grape skins. They quantitatively analyzed the polyphenolic content of red grape skin extract and found a high concentration of malvidin. Using isolated rat hearts, they observed that the extract and malvidin induced positive effects on heart contractions, relaxation, and coronary dilation. The mechanism of action involved the activation of the PI3K/NO/cGMP/PKG pathway, leading to increased levels of intracellular cGMP and the phosphorylation of various proteins involved in cardiovascular regulation. They also discovered that malvidin exhibited cardioprotective properties against ischemia/reperfusion damage. These findings contribute to understanding the cardiovascular benefits of food-derived polyphenols and highlight malvidin as a potential cardioprotective compound, which is significant for nutraceutical research (67–72).

### 2.8. Bioflavonoid extraction techniques

There are several ways employed to extract bioflavonoids from different sources (73). Yao and colleagues reported the extraction of 21 bioflavonoids using ultra-high-performance liquid chromatography and rapid microwave extraction in tandem with a triple quadrupole-linear ion trap mass spectrometry (1). Mangang et al. (74) extracted bioflavonoids from the bark of *Albizia myriophylla* using a microwave-assisted method. Chahyadi & Elfahmi examined the impact of different extraction techniques on rutin yield from cassava leaves. The duo extracted rutin from the leaves using microwave-assisted extraction (MAE), ultrasound-assisted extraction (UAE), reflux, boiling, and maceration. Manzoor and colleagues reviewed different methods of luteolin extraction like UAE, MAE, soxhlet, maceration, reflux, ultrasound microwave-assisted extraction, supercritical fluid extraction (SFE), high-speed counter-current chromatography extraction techniques, hydrodistillation, and enzyme-assisted extraction. Needless to say, each and every extraction technique has pros and cons (74–77).

## 3. Cryopreservation techniques for bioflavonoid-rich plant sources

Scientists employ cryopreservation, a conservation technology in which ultra-low temperatures (−135°C to −196°C) are used to preserve plant cells and tissues for quite a long time with very slim chances of variation occurrence. Generally, there are many reasons if not advantages for cryopreserving plant sources. These are (a) conservation of endangered species, (b) elimination of virus-infected plant species by employing shoot apex cryotherapy, and (c) minute storage requirements among others. Scientists are studying and trying to optimize cryopreservation techniques. They are cryopreserving bioflavonoid-rich sources due to their beneficial health properties. The cryopreservation technique is daunting. There are principles and different cryopreservation techniques (11).

Cryopreservation is utilized for various reasons, including the facilitation of crossbreeding between plants that have different flowering periods and those located in distant regions. It also helps in reducing the transmission of diseases through pollination vectors and enables the long-term preservation of superior germplasm. By employing cryopreservation, the risk of encountering variations, such as somaclonal variation in tissue culture or gametoclonal variation in field collections, is minimized. Consequently, it becomes feasible to acquire cultures with genetically uniform clones that can be propagated vegetatively (91).

Researchers reported many novel cryoprotectants (CPAs). In 1979, three scientists reported proline as a novel CPA for the storage of *Zea mays* cultured cells in liquid nitrogen. Proline-cryopreserved cells exhibit higher recovery potential, reduced loss of viability after thawing, and increased tolerance to various post-thaw culture conditions as compared to glycerol and dimethylsulfoxide. The mechanism of action of proline is proposed to be similar to its role in protecting cells against natural stresses. It is suggested that proline may safeguard cells against solution effects caused by dehydration during freezing. Trehalose, as another novel CPA, was reported in 1985 when a research group cryopreserved carrot and tobacco cells. One of their findings was that the highest level of viability following thawing (71-74%), as determined by phenosafranin dye exclusion, was achieved when the carrot cells were treated with either 5% or 10% trehalose for 24 h prior to freezing, using 40% trehalose as the cryoprotectant. Trehalose’s effectiveness as a CPA is believed to stem from its ability to interact with lipid membranes and safeguard them, stabilize proteins when subjected to freezing and thawing, and create a solid and stable glass-like structure to preserve cells during the cooling process (92–94).

### 3.1. Vitrification

**Figure 2:**
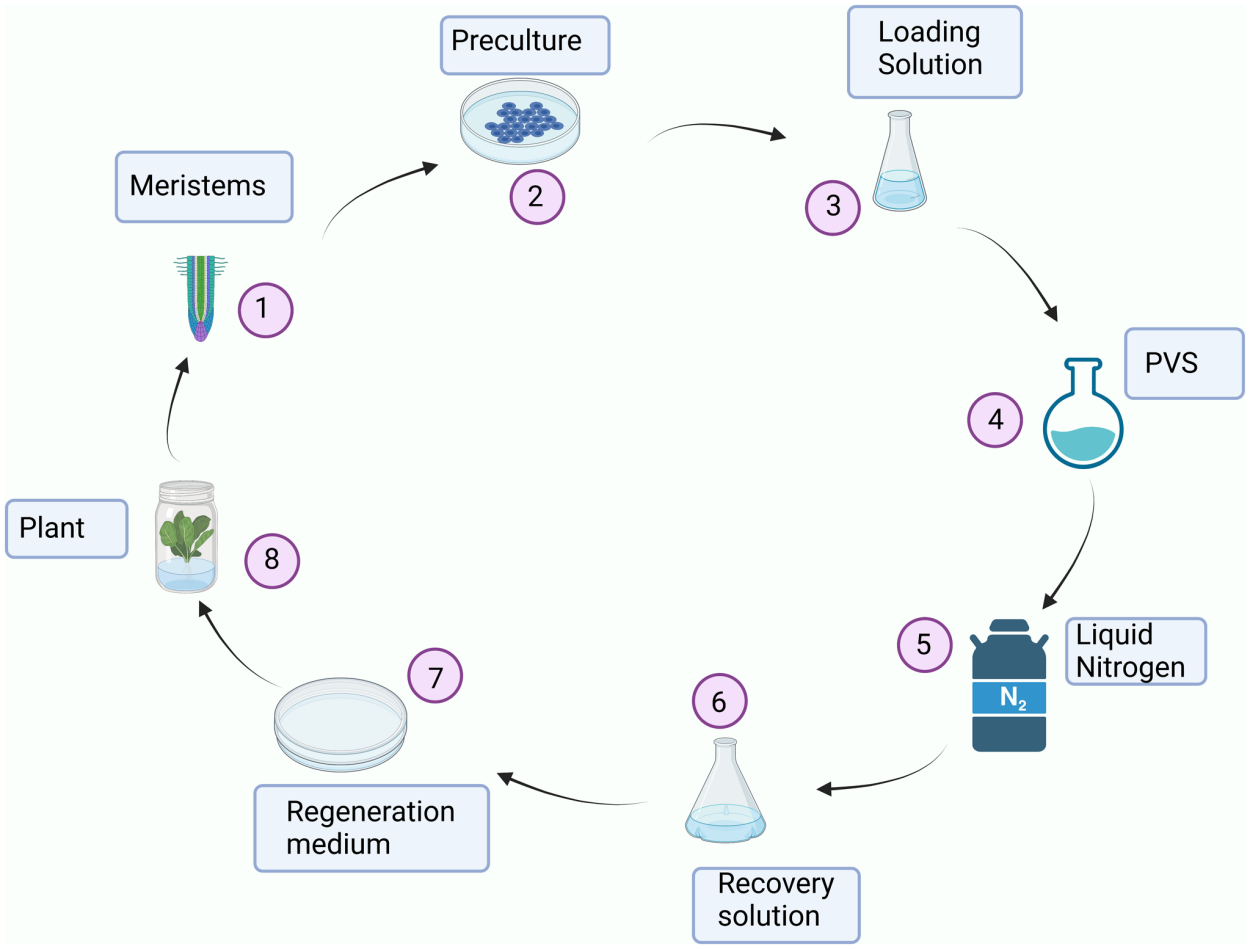
An overview of vitrification of plant cells. The cells to be cryopreserved are obtained from actively dividing parts of a plant like apical or root meristems (1). The cells are precultured (2). The cells are then transferred to a loading solution (3). After that, the cells are put in a PVS (4). The cells are then plunged into liquid nitrogen (5). Now, for thawing purposes, the cells are put in a recovery solution (6). Regeneration medium (7) is needed for the cells to develop into a plant (8).

Vitrification (Figure 2) is a technique that allows glass state formation without crystallization. This is one of the major cryopreservation principles. Scientists employ this technique to preserve plants. However, the major challenge is that most plant species have high water content and/or lack glassy state-inducing substances. Such plants do not have the ability to vitrify naturally. Only a few plants can vitrify naturally. Cryoprotectants used during the vitrification must have at least three principal properties: (a) minimal toxicity to the plant cells, (b) high glass-sate forming capacity, and (c) sufficient dehydration strength to vitrify the plant cells among others. The commonly used cryoprotectants used are glycine, dimethyl sulfoxide (DMSO), ethylene glycol, propylene glycol, sucrose, and sorbitol in this order. The concentrations vary though. There are different versions of vitrification techniques like encapsulation-vitrification, and droplet-vitrification (14, 95).

Scientists came up with plant vitrification solutions (PVS), which are chemicals mixed in different proportions to optimize the vitrification technology. These solutions are used in modern plant cryopreservation techniques because of their protective effects and their osmotic characteristics. The PVS solutions increase viscosity at lower temperatures, leading to an amorphous glassy state, without forming ice. The common four PVS solutions are PVS1, 2, 3, and 4. It is reported that Uragami pioneered the use of PVS1(0.06 DMSO, 0.22 Glycine, 0.13 Ethylene glycol, and 0.13 Propylene glycol) to culture cells derived from *Asparagus officinalis* L mesophyll tissue. We suggest one can look at an article written by Zamecnik et al. (14) for a better understanding of PVS. The solutions come into contact with the cell and then form PVS solute compounds changing the glass transition temperature, and/or with the bound water, which hinders the intercellular ice crystals-formation (14, 96–99).

Osmoprotective solutions are concocted by mixing non-penetrating and low or moderately-concentrated penetrating cryopreservants. In plant cryobiology, the most used osmoprotective solution is known as loading solution 1 (LS1). This solution is a mixture of 0.4 M sucrose and 2.0 M glycerol. Other loading solutions are composed of ethylene glycol, DMSO, sucrose, and glycerol in different combinations and concentrations. As such, there is LS2, 3, and 4. The purpose of the LS is to prepare the plant specimens biophysically to osmotic stress by exposure to a moderately concentrated osmoticum, prior to severe dehydration with highly concentrated cryoprotectants (CPAs) (91, 100).

### 3.2. Slow freezing technique

The slow freezing technique involves subjecting plant cells to a mixture of membrane-permeating cryopreservants (pCPAs) and non-cell membrane permeating CPAs (npCPAs) before cooling at a controlled rate to an average low temperature and lastly plunging the vials into liquid nitrogen. Scientists report that the pCPAs prohibit intracellular ice formation whereas npCPAs cause net efflux of water from the cells. The use of one type of CPA mitigates post-thaw regrowth rates to less than 30%, whereas both types in concert scored more than 60% regrowth as demonstrated for wheat (*Triticum aestivum*) and brome (*Bromus inermis*) cells. In spite of the protective properties of the CPAs, the direct plant cell freezing in liquid nitrogen has not yet so far resulted in their regrowth following thawing. This implies that an intermediate cooling step between –25°C and –40°C is mandatory. The commonly used cooling rate is – 0.2°C min^–1^ (91, 101–107).

### 3.3. Factors affecting plant cryopreservation

Scientists argue that the success of plant cryopreservation depends on factors like the origin of plant cell cultures (PCCs) and the balance between the mixtures of pCPAs and npCPAs among others. PCCs are typically sourced from plants through an intermediate callus. The callus is an unorganized mass of dedifferentiated plant cells that form if excised tissues (explants) are subjected to a mixture of auxins and cytokinins *in vitro*. Auxins prohibit organogenesis whereas cytokinins promote cell division. As a result, the presence of these two phytohormones promotes continuous cell division without cell differentiation. A callus is normally generated from flowers, leaves, petioles, and roots. Seedlings, buds, and young leaves are not conducive to generating a callus because they contain actively dividing cells called meristems. In one research, the *Gentiana tibetica* PCCs were prepared in a 0.4 M sucrose CPA for a week. The follow-up protocol employed was vitrification. The scientists observed that the post-thaw viability of the cells was 90%. In a *Gentiana. tibetica* generated PCCS that were subjected to 0.4 M sorbitol for 2 days, a follow-up technique was slow freezing. The post-thaw viability of these cells was found to be 3%. It was also observed that adjusting cell density properly resulted in plant growth following cryopreservation. There is what is referred to as the minimum cell density threshold which is the cell density that results in plant growth following cryopreservation. The most common packed cell volumes (PCVs) for slow freezing and encapsulation-dehydration techniques used are 20-30%. These PCVs yield around 80% plant growth following cryopreservation. The usual PCVs for the vitrification technique are within the range of 40–50% (107).

### 3.4. Impact of cryopreservation on bioflavonoid content and bioactivity

Different preservation techniques have different impacts on plants’ content and bioactivity of bioflavonoids. In one study, after cryopreservation, the antioxidative activity of two *Hypericum* species that have been grown *in vitro* namely *H. rumeliacum Boiss*. and *H. tetrapterum* Fr. was estimated. Following a vitrification-based cryopreservation procedure, both species were successfully regenerated. In response to cold temperatures, flavonoids assemble in leaves and stems. Cold stress is said to boost the biosynthesis of flavonoids, according to recent reports. Additionally, flavonoid compounds were primarily blamed for some of the beneficial qualities of *Hypericum species*. The increased flavonoid production in the two experimental species is an unanticipated positive outcome of cryopreservation. In *H. tetrapterum* tissues, there was a 237% increase in flavonoid content compared to the control. Total antioxidant activity was found to vary between control values in cryopreserved *H. tetrapterum* while increasing (33% above control) in regenerated *H. rumeliacum* plants, which showed the presence of oxidative stress and a lower tolerance for cryopreservation (108).

In another investigation into the effects of various drying methods, including freeze-drying and hot air oven drying, on flavonoids, specifically, flavanone glycosides, antioxidant potential, and total phenol content in immature dropped fruits of *Citrus reticulata* Blanco, bioactive compounds were identified and quantified. Using high-performance liquid chromatography (HPLC), flavonoids were measured. Azino-bis [3-ethylbenzthiazoline-6-sulfonic acid] (ABTS), 2,2-diphenyl-1-picrylhydrazyl radical (DPPH), the ferric reducing ability of plasma (FRAP), and total phenol concentration were used to test the antioxidant activity. In comparison to hot air-dried samples (17.99%), freeze-dried samples of 12- and 14-mm size retained the highest amounts of hesperidin flavonoid content (27.03 and 27.20%) and higher phenolic content, which ranged from 50.54 to 54.19 mg GAEL^–1^ (109).

## 4. Microencapsulation techniques for bioflavonoid-rich sources

Active molecules or compounds like vitamins, pigments, antimicrobials, and flavorings, among others, are shielded from the environment using modern microencapsulation processes. Microencapsulation has benefits including easier handling and control over the release and solubilization of active ingredients, which makes it a fantastic area for advancement in food science and processing. For instance, the usage of microcapsules in various food matrices such as meat products, dairy products, cereals, and fruits, as well as in their derivatives, may be effective in the production of functional food items, fat reduction, sensory improvement, preservation, and other areas. The variety of processes and materials utilized in the microencapsulation process leads to the adaptability of applications. Examples of microencapsulation techniques are spray drying, spray chilling, spray cooling, fluidized bed coating, liposome entrapment, extrusion, freeze-drying, and coacervation (110).

### 4.1. Freeze-drying

**Figure 3:**
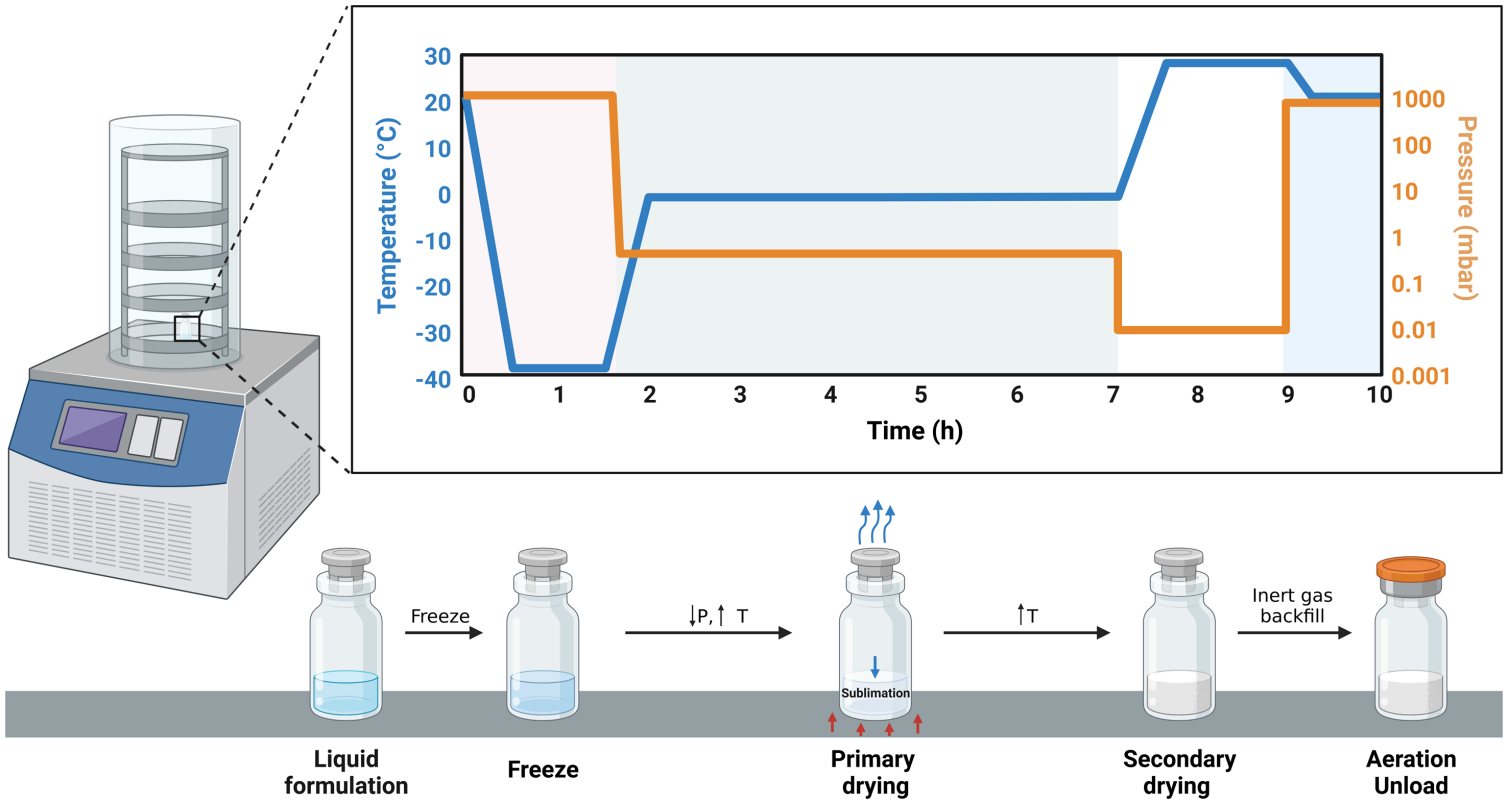
Schematic illustration of the freeze-drying process. In the first phase (01–1.5 h), the sample is frozen as the temperature falls from 20 to –40°C whereas pressure is constant (1,000 mbar). The second phase (2–7 h) is characterized by a sharp drop in pressure and a sharp increase in temperature leading to the sublimation of the solvent (primary drying).Finally, secondary drying takes place resulting in an encapsulated product.

Freeze-drying (Figure 3) is a widely used method of encapsulation that involves dehydrating a frozen sample through sublimation. It is particularly suitable for preserving sensitive bioactive compounds since it does not subject the substances to high temperatures like spray-drying. Freeze-dried products can be quickly and easily reconstituted, making them valuable for emergency situations such as administering antibodies and vaccines promptly. Additionally, the freeze-drying technique is simpler compared to other microencapsulation methods, as it involves a limited number of steps (111).

The encapsulation process is crucial for maintaining the stability of freeze-dried powders, and the choice of coating or carrier materials plays a significant role in its efficiency and physicochemical properties. These materials encompass the active core materials during microencapsulation, and they can be sourced from natural or synthetic options, such as maltodextrins, modified starches, proteins, dextrins, among others. The encapsulating agents’ selection is vital in ensuring the overall effectiveness of the process and the preservation of the encapsulated substances. Milea and colleagues investigated the encapsulation efficiency of anthocyanins extracted from sweet cherry skins using two different wall materials, namely whey protein isolate (WPI) and chitosan. The study found that the encapsulation efficiency was approximately 77.68% with a narrow variation of ±2.57%. Comparing this to a similar study by Oancea et al., who worked with sour cherry skins, the encapsulation efficiency of anthocyanins microencapsulated in WPI and acacia gum was reported to be 70.28% with a standard deviation of ±2.17%. Furthermore, when a single protein was used as the wall material, the encapsulation efficiency ranged from 44.79 to 64.69% as reported by Mamani-Matsuda et al. In a separate investigation conducted by Stănciuc et al. with grape skin extract, the encapsulation efficiency was found to be remarkably higher, reaching 94% when using acacia gum and 99% when employing pectin as the wall material. These findings highlight the influence of both the fruit source and the choice of wall material on the encapsulation efficiency of anthocyanins, which are valuable natural compounds with potential health benefits (112–115).

In one study, scientists aimed to determine if phenolic compounds from *Vaccinium species* leaves, both as microencapsulated powder and aqueous extracts, are bioaccessible after being exposed to *in vitro* simulated digestion. The effect of maltodextrin and glucose microencapsulation carriers on the phenolic content of the extracts was also evaluated. Prior to encapsulation, the emulsions’ viscosity was demonstrated at a shear stress of 50 s^-1^ dilatant, and above this point, they exhibited Newtonian behavior with a final viscosity ranging between 1.024 and 1.049 mPas. The samples’ final microencapsulation yield varied from 79 to 81%. The phenolic content dropped following gastrointestinal digestion, despite the microencapsulated forms offering a targeted release at the intestinal level. The microencapsulated extracts’ bioaccessibility displayed greater values than those of their non-encapsulated counterparts (116). Many other studies are also conducted on the microencapsulation of bioflavonoids (10, 111, 117–127).

## 5. Cryopreservation of microcapsules that contain bioflavonoids

Bioflavonoids are frequently used in different medicinal and cosmetic formulations as well as utilized as nutritional supplements. Cryopreserving bioflavonoid-containing microcapsules can be advantageous for a number of reasons. Microcapsules may be kept for a very long time by being cryopreserved, which guarantees that the bioflavonoid content will do so too. This is especially helpful when a lot of microcapsules need to be preserved for a long time for research, production, or distribution purposes. In addition, due to their sensitivity to heat, light, and oxygen, bioflavonoids may degrade and lose some of their therapeutic characteristics. The bioactivity of the encapsulated bioflavonoids is preserved via cryopreservation at extremely low temperatures, which reduces degradation. Furthermore, compared to other preservation techniques, cryopreserved microcapsules can be more conveniently packed, stored, and transported at lower costs. This enables the microcapsules containing bioflavonoids to be distributed and used with greater flexibility (13, 14).

Cryopreserving bioflavonoid-containing microcapsules has a number of benefits and potential uses in the nutraceutical and functional food industries. Some of the potential advantages and uses are tabulated below (Table 2).

**Table 2:** The benefits and potential applications in functional foods and nutraceuticals.

Health benefits	Integration of cryopreserved microcapsules containing bioflavonoids into functional foods and nutraceuticals can have: Antioxidant effects that are cardio-protective, maintaining skin health Immunomodulatory effects that boost the immune system, anti-cancer properties Anti-inflammatory effects	(108, 120)
	Combination with Other Ingredients To generate synergistic formulations with improved health benefits, cryopreserved microcapsules can be mixed with other active components, such as vitamins, minerals, or probiotics.	(33)
	Enhanced Bioactivity Bioflavonoids can be shielded by microencapsulation from environmental variables including light, heat, and oxygen that can cause them to degrade, maintaining their bioactivity and increasing their health advantages.	(128)
	Controlled Release A regulated release of bioflavonoids from microcapsules can improve their bioavailability and therapeutic benefits by enabling targeted administration and absorption in the body.	(33, 128)
Economic benefits for the neutraceutical and food industries	Enhanced Stability By conserving the chemical makeup and biological activity of bioflavonoids during storage and transportation, cryopreservation aids in maintaining their stability and integrity.	(33, 128)
	Personalized Nutrition Microencapsulation’s flexibility enables the modification of bioflavonoid formulations, enabling individualized dietary regimens and customized functional food and nutraceutical products. Improved Taste and Texture When used in functional food and nutraceutical formulations, microcapsules may cover up the taste and odor of bioflavonoids, making them more acceptable to consumers.	(104)
	Extended Shelf Life The shelf life of bioflavonoids in functional foods and nutraceutical products can be extended through microencapsulation and cryopreservation, preserving their potency and usefulness for a longer time.	(33, 128)

### 5.1. Microencapsulation improves bioflavonoids’ shelf-life

Environmental conditions and microbial attacks are the two major challenges that affect bioflavonoids’ shelf-life in the food industry. Scientists report that cryopreserving and encapsulating the bioflavonoids extend their shelf-life. For example, Saqueti and colleagues conducted an experiment to investigate the shelf-life of cryopreserved and microencapsulated bioactive compounds from Acerola pulp over a period of 6 months. The findings demonstrated that microencapsulation was more effective in retaining the antioxidants present in Acerola pulp compared to other preservation methods utilized. This was attributed to the higher concentration of bioactive compounds (BCPs) within the microencapsulated form and the subsequent decrease in pH. Moreover, the results indicated that microencapsulation outperformed lyophilization in preserving the desired compounds in Acerola pulp over a span of 6 months, as evidenced by the superior BCPs content in the microencapsulated samples. These results highlight the potential of microencapsulation as an attractive option for the food industry (129, 130).

## 6. Challenges and future in cryopreservation and microencapsulation of bioflavonoid-rich sources

There are stability problems with bioflavonoids. Because of this, their potency and bioavailability are lowered during cryopreservation and storage. In addition, the process of freezing and thawing can harm cells, which compromises the structural and metabolic stability of bioflavonoids. The same is true for sources of bioflavonoids that have been cryopreserved. Furthermore, scientists observed that some bioflavonoids are difficult to encapsulate and preserve because of their poor solubility in aqueous solutions (131).

## 7. Future perspectives

Future research should focus on creating novel cryoprotectants that reduce cryoinjury while improving the stability of bioflavonoids during freezing and thawing processes. Also, there is a need to explore cutting-edge microencapsulation techniques like spray drying, electrostatic encapsulation, or nanoemulsion technology to boost encapsulation effectiveness and safeguard bioflavonoids. Setting up strong quality control procedures and standardized protocols for cryopreservation and microencapsulation procedures to guarantee constant product efficacy and quality is of paramount importance. Furthermore, enhancing bioavailability entails looking into methods for increasing the bioavailability of encapsulated bioflavonoids, including enhancing absorption, enhancing release kinetics, and reducing metabolism in the body. Another area of research that requires improvement is the investigation of bioflavonoid combinations with other natural substances or excipients that can improve their stability, solubility, and overall therapeutic effects. New synergistic formulations are produced as a result. Scientists have another future consideration regarding this area of research. Fruits and vegetables, which are sources high in bioflavonoids, frequently have palatable sensory qualities including taste, color, and scent. For products to be accepted by consumers, these sensory qualities must be preserved after cryopreservation and microencapsulation. The creation of encapsulation materials and procedures that reduce changes in sensory qualities during storage and subsequent reconstitution may be one future option. Finally, there is a need to address regulatory challenges associated with the use of cryopreserved and microencapsulated bioflavonoids, including safety, efficacy, and labeling requirement (110, 116, 127, 131).

## 8. The other side of bioflavonoids

**Figure 4:**
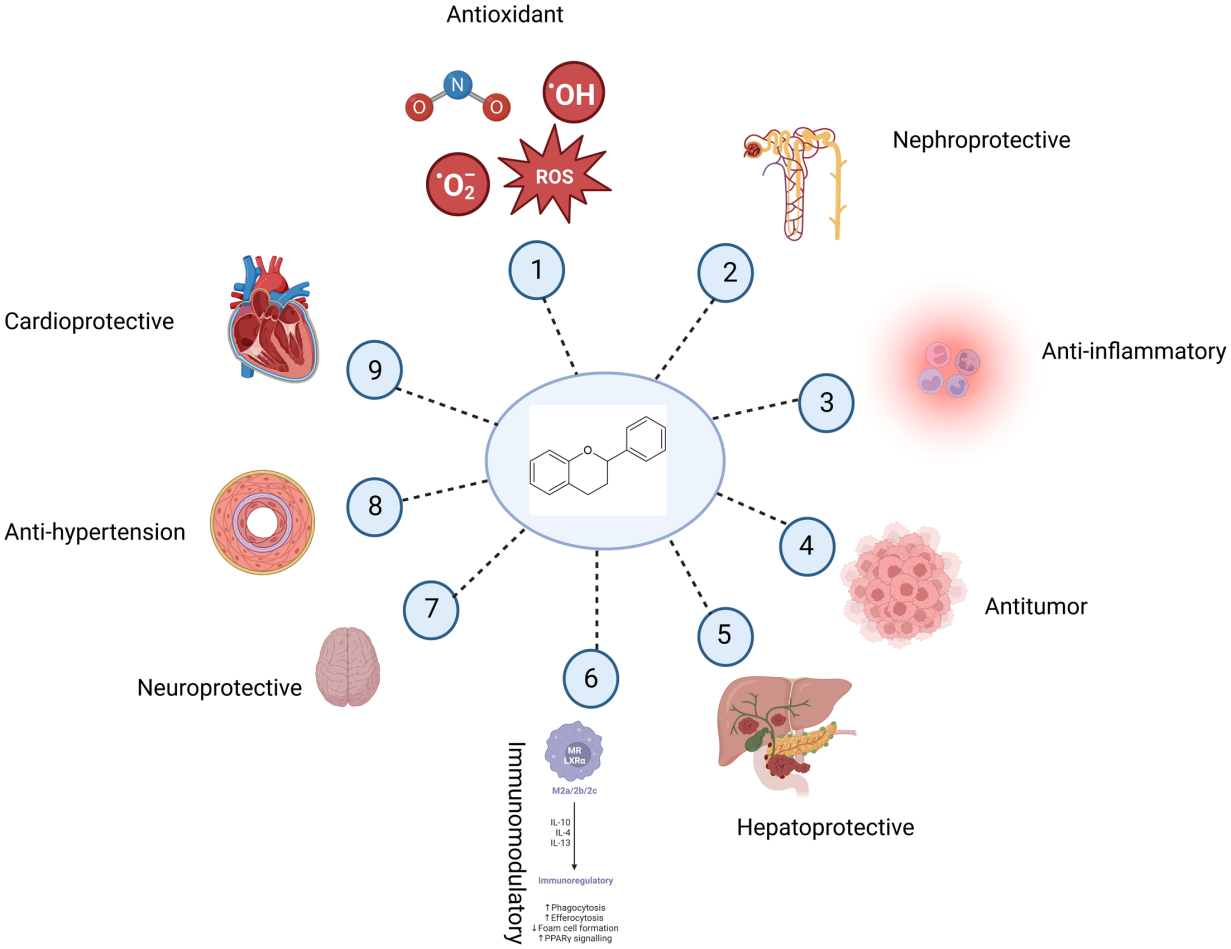
Potential health benefits of bioflavonoids. Bioflavonoids have antioxidant (1), nephroprotective (2), anti-inflammatory (3), anticancer (4), hepatoprotective (5), immunomodulatory (6), neuroprotective (7), anti-hypertension (8), and cardioprotective (9) properties.

A lot of papers focus on the positive benefits of polyphenolic compounds, bioflavonoids in particular (Figure 4). As such, bioflavonoid supplements are on sale. Nevertheless, there are research articles that highlight the negative impacts in almost all areas of the potential therapeutic effects of these compounds. This section is going to focus on the other side of bioflavonoids’ antitumor activities. Genistein can stimulate the growth of MCF-7 cells, which are dependent on estrogen, at low concentrations while acting as a cytostatic agent at higher doses, according to earlier research (132, 133). These results were in line with those of an additional animal study in which mice with ovariectomies were implanted with MCF-7 cells. The scientists’ hypothesized mechanism is most likely caused by genistein’s connection to the MCF-7 cells’ estrogen receptor pathway. A study on animals revealed that genistein consumption at a daily dose of 250 mg/kg enhanced the aggressive progression of prostate cancer in transgenic adenocarcinoma mouse prostate mice, but that genistein consumption at a higher dose of 1,000 mg/kg did not have the same effect (62). The mechanism suggested that it could enhance the proliferative and metastatic early-stage prostate cancer in an estrogen and phosphatidylinositol 3 kinases (PI3K)- dependent pattern. It was also found that daidzein also influenced the proliferation of T-47D breast cancer cell lines in biphasic dose-dependent ways (134).

Additionally, it has been noted that quercetin stimulates cell proliferation in a dose-dependent manner. The human breast cancer cell lines MCF-7 SH and MCF-7 WT proliferated more when quercetin was present in low quantities (Q (22).) The reactive metabolites of quercetin oxidation, which affect the synthesis of DNA and the ER-dependent pathway, are the potential mechanism according to the *in vitro* studies conducted (135). This is just but a tip of an icebag concerning the other side of bioflavonoids. Therefore, it is imperative that thorough research is done along these lines.

## 9. Conclusion

To conclude, cryopreservation and microencapsulation of bioflavonoids seem to be the best methods ever available currently since bioflavonoids’ shelf-life, bioavailability, and solubility are improved. Encapsulation can mask the unpleasant odor of the bioflavonoids as nutraceuticals. Cryopreservation is utilized for various reasons, including the facilitation of crossbreeding between plants that have different flowering periods and those located in distant regions. It also helps in reducing the transmission of diseases through pollination vectors and enables the long-term preservation of superior germplasm. By employing cryopreservation, the risk of encountering variations, such as somaclonal variation in tissue culture or gametoclonal variation in field collections, is minimized. Consequently, it becomes feasible to acquire cultures with genetically uniform clones that can be propagated vegetatively There are major challenges faced by the scientists when employing these two technologies, however. The CPAs can damage the cells’ integrity. In addition, freeze-drying, which is the most favorable encapsulation technique is costly. Scientists are in search of novel CPAs that mitigate cell damage during cryopreservation. Also, they are exploring new encapsulation technologies.

## Author contributions

JX and RM: Wrote the paper. IS, YH, JK, YS, and HS: Participated in the design and drafting of this manuscript. WZ, BH, and ST: Guided the writing of the paper and reviewed the manuscript. All authors critically reviewed progressive drafts of the manuscript and approved the final version.

## Funding

This research was supported by Changsha Medical University.

## Conflict of interest

The authors declare that the research was conducted in the absence of any commercial or financial relationships that could be construed as a potential conflict of interest.

## Publisher’s note

All claims expressed in this article are solely those of the authors and do not necessarily represent those of their affiliated organizations, or those of the publisher, the editors and the reviewers. Any product that may be evaluated in this article, or claim that may be made by its manufacturer, is not guaranteed or endorsed by the publisher.
